# TLR8 regulation of *LILRA3* in monocytes is abrogated in human immunodeficiency virus infection and correlates to CD4 counts and virus loads

**DOI:** 10.1186/s12977-016-0248-y

**Published:** 2016-03-12

**Authors:** Hui Zhi Low, Gerrit Ahrenstorf, Claudia Pommerenke, Nadine Habermann, Klaus Schughart, David Ordóñez, Renata Stripecke, Esther Wilk, Torsten Witte

**Affiliations:** Department of Clinical Immunology and Rheumatology, Hannover Medical School, Carl-Neuberg-Str. 1, 30625 Hannover, Germany; Department of Hematology, Hemostasis, Oncology and Stem Cell Transplantation, Hannover Medical School, Hannover, Germany; Department of Infection Genetics, Helmholtz Centre for Infection Research, Braunschweig, Germany; University of Veterinary Medicine, Hannover, Germany; University of Tennessee Health Science Center, Memphis, TN USA

**Keywords:** LILRA3, TLR8, TLR4, Monocytes, HIV

## Abstract

**Background:**

LILRA3 is an immunostimulatory molecule which can conditionally induce the proliferation of cytotoxic cells. *LILRA3* has a deletion genotype which is associated with multiple immune disorders. In this study, we wanted to analyze the regulation of *LILRA3* and its significance in the context of HIV infection.

**Results:**

We analyzed a panel of TLR agonists and found that ssRNA40, a TLR8 agonist, is a potent inducer of *LILRA3* in healthy individuals. However, this regulation is much diminished in HIV. Comparison of TLR8 to TLR4 induction of LILRA3 indicated that LPS induces less *LILRA3* than ssRNA40 among healthy controls, but not HIV patients. Levels of *LILRA3* induction correlated to virus load and CD4 counts in untreated patients. Recombinant LILRA3 can induce a host of proinflammatory genes which include IL-6 and IL-1α, as well as alter the expression of MHC and costimulatory molecules in monocytes and B-cells.

**Conclusion:**

Our experiments point towards a beneficial role for LILRA3 in virus infections, especially in ssRNA viruses, like HIV, that engage TLR8. However, the potentially beneficial role of LILRA3 is abrogated during a HIV infection. We believe that more work has to be done to study the role of LILRA3 in infectious diseases and that there is a potential for exploring the use of LILRA3 in the treatment of virus infections.

**Electronic supplementary material:**

The online version of this article (doi:10.1186/s12977-016-0248-y) contains supplementary material, which is available to authorized users.

## Background

Extensive work has been done to functionally characterize members of the leukocyte immunoglobulin like receptor (LILR) family [1–11]. In comparison, the function of leukocyte immunoglobulin like receptor A3 (LILRA3; ILT6; CD85e) has been less well characterized. LILRA3 is a 70 kDa highly glycosylated protein secreted by monocytes. The differentiation of monocytes into osteoclasts and dendritic cells has been shown to upregulate LILRA3 [12, 13]. It is the only member of the LILR family that exists constitutively as a soluble protein due to a lack of a transmembrane domain and cytoplasmic tail. LILRA3 binds to HLA-G and classical HLAs with a much lower affinity than LILRB1 and LILRB2 [14], although it seems to bind to free heavy chain forms of HLA-C with higher affinity [15].

Around 30 % of healthy Caucasians carry the *LILRA3* deletion of 6,7 Kb which includes nearly the complete coding sequence [8, 16]. The homozygous deletion is found in 3 % of the healthy Caucasian population and confers susceptibility to some autoimmune diseases [17–19], HIV-infection (in revision), and B cell non-hodgkin lymphoma [20].

Other members of the LILR family were demonstrated to play important roles in HIV infection [21]. Myeloid dendritic cells in elite controllers, who spontaneously maintain low viremia, have a selective upregulation of LILRB1 and LILRB3, whereas patients with a progressive infection showed a downregulation of LILRA2 and upregulation of LILRB2 [22].

As we have shown in a previous report, LILRA3 is an immunostimulatory molecule that specifically induces the proliferation of CD8 T-cells and NK-cells in the presence of allogeneic stimulation, as well as the production of pro-inflammatory cytokines [20]. Based on these properties, we suspect that LILRA3 could be involved in immune responses against viruses. However, nothing is known about the processes upstream of LILRA3 and their regulation.

Pattern recognition receptors (PRRs) are widely expressed on a variety of immune sensing cells. They recognize pathogen associated molecular patterns to initiate the innate immune response and direct adaptive immunity [23, 24]. The best characterized PRRs are the toll-like receptors (TLR). Among the 10 members which sense a variety of infection types, those associated with virus infections are intracellular TLR3 (dsRNA), -7, -8 (both ssRNA) and -9 (unmethylated CpG DNA).

We used TLR-agonists as surrogates for whether virus infections could induce expression of *LILRA3*. We wanted to determine which TLR agonist could regulate *LILRA3* expression and showed that ssRNA40, a TLR8 agonist, is a most prominent regulator of *LILRA3*. Since HIV is an ssRNA virus which has been documented to induce aberrant TLR responses in hosts [25, 26], we analyzed the TLR8 regulation of *LILRA3* expression in the context of HIV infection.

## Results

### TLR-8 induces *LILRA3* expression in CD14^+^ monocytes

**Fig. 1:**
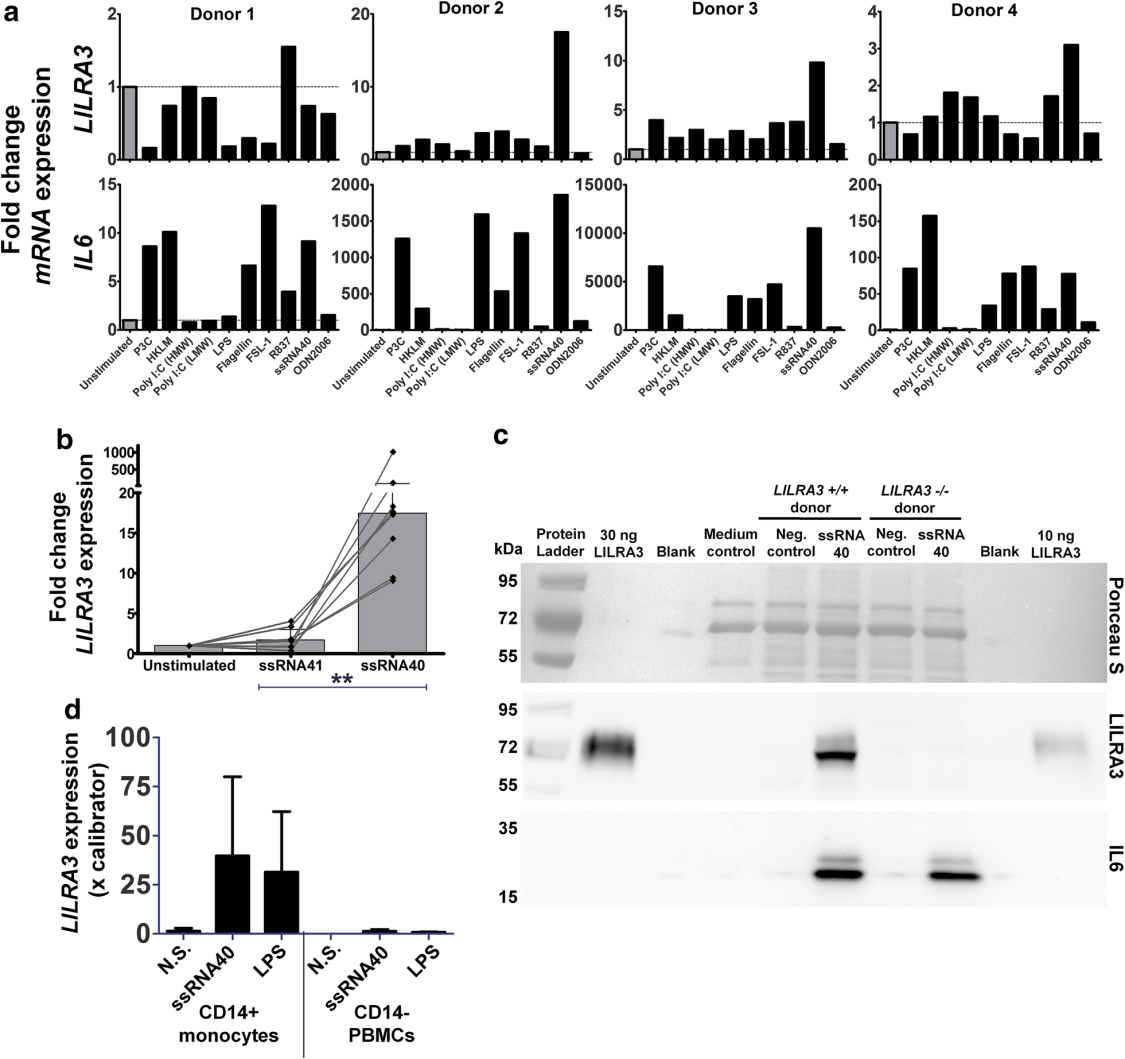
LILRA3 induction by TLRs. **a** Induction of *LILRA3* and *IL6* expression by a panel of TLR agonists. *LILRA3* and *IL6* expression was measured, using qPCR, as fold change to the unstimulated control from PBMCs stimulated for 24 h with Pam3CSK4 (P3C), heat killed *Listeria monocytogenes* (HKLM), polyinosinic-polycytidylic acid (Poly I:C) in high molecular weight (HMW) and low molecular weight (LMW) forms, lipopolysaccharide (LPS), flagellin, synthetic diacylated lipoprotein FSL-1, Imiquimod (R837), ssRNA40/LyoVec and CpG oligonucleotide ODN 2006. **b** Induction of *LILRA3* by ssRNA40 on the transcriptional level. *LILRA3* expression was measured as fold change to the unstimulated control from PBMCs stimulated for 24 h with uridine-rich HIV-derived ssRNA40, using ssRNA41 as control (n = 8). Wilcoxon matched-pairs signed rank test was used to compare the median between ssRNA41 and ssRNA40 stimulated *LILRA3* expression. Scatter dot plot overlayed on bar graph displayed as median with interquartile range. **c** Induction of *LILRA3* in PBMCS by ssRNA40 on the protein level. 30 × 10^6^/mL PBMCs from *LILRA3* ^+*/*+^ and *LILRA3* ^−*/*−^ donors were stimulated with 5 μg/mL ssRNA40. 40 µL from each supernatant was loaded and Ponceau S staining was used as loading control. The membrane was blotted for LILRA3 and IL6, using recombinant LILRA3 as protein control. **d** Expression of ssRNA40-induced *LILRA3* by monocytes. PBMCs stimulated overnight with ssRNA40 and LPS were purified for CD14^+^ monocytes using magnetic-activated cell sorting. The resulting CD14^+^ positive and depleted fractions were analysed via qPCR for *LILRA3* expression, normalized to *RPLP0* and relatively quantified to calibrator cDNA (*p < 0.05; **p < 0.01)

In preliminary experiments, we subjected PBMCs from four donors to a panel of TLR agonist. Of the nine TLR agonists tested, a consistent immune response among the four donors, measured using IL6 expression, was only observed for Pam3Csk, heat-killed Listeria monocytogenes, LPS, flagellin, FSL-1 and ssRNA40. Three of the four donors had a substantial upregulation of *LILRA3* to TLR8 agonist ssRNA40 (Fig. 1a), but we did not observe any obvious pattern of upregulation in *LILRB1* and *LILRA1* expression (Additional file 1: Figure S1). In order to confirm that TLR8 stimulation significantly upregulates *LILRA3*, we expanded the cohort using ssRNA40 stimulation, with ssRNA41 (similar to ssRNA40, but with uridine replaced with adenosine) as a control. Whereas ssRNA40 significantly upregulated the expression of *LILRA3*, ssRNA41 did not (Fig. 1b). LILRA3 could be detected in the supernatant of ssRNA40 stimulated *LILRA3* positive PBMCs, but not in the negative controls and in the *LILRA3*^−*/*−^ donor, even though both donors were positively activated by ssRNA40 to secrete IL6 (Fig. 1c).

We determined monocytes to be the major producer of *LILRA3* under ssRNA40 stimulation, as *LILRA3* mRNA was detected only in CD14^+^ monocytes after 22-h ssRNA40 stimulation in PBMCs. In all three donors tested, almost all of the *LILRA3* expression occurred in CD14+ monocytes, with two of the three donors expressing higher *LILRA3* under ssRNA40 stimulation compared to LPS (Fig. 1d).

### *LILRA3* is upregulated after other proinflammatory cytokines

**Fig. 2:**
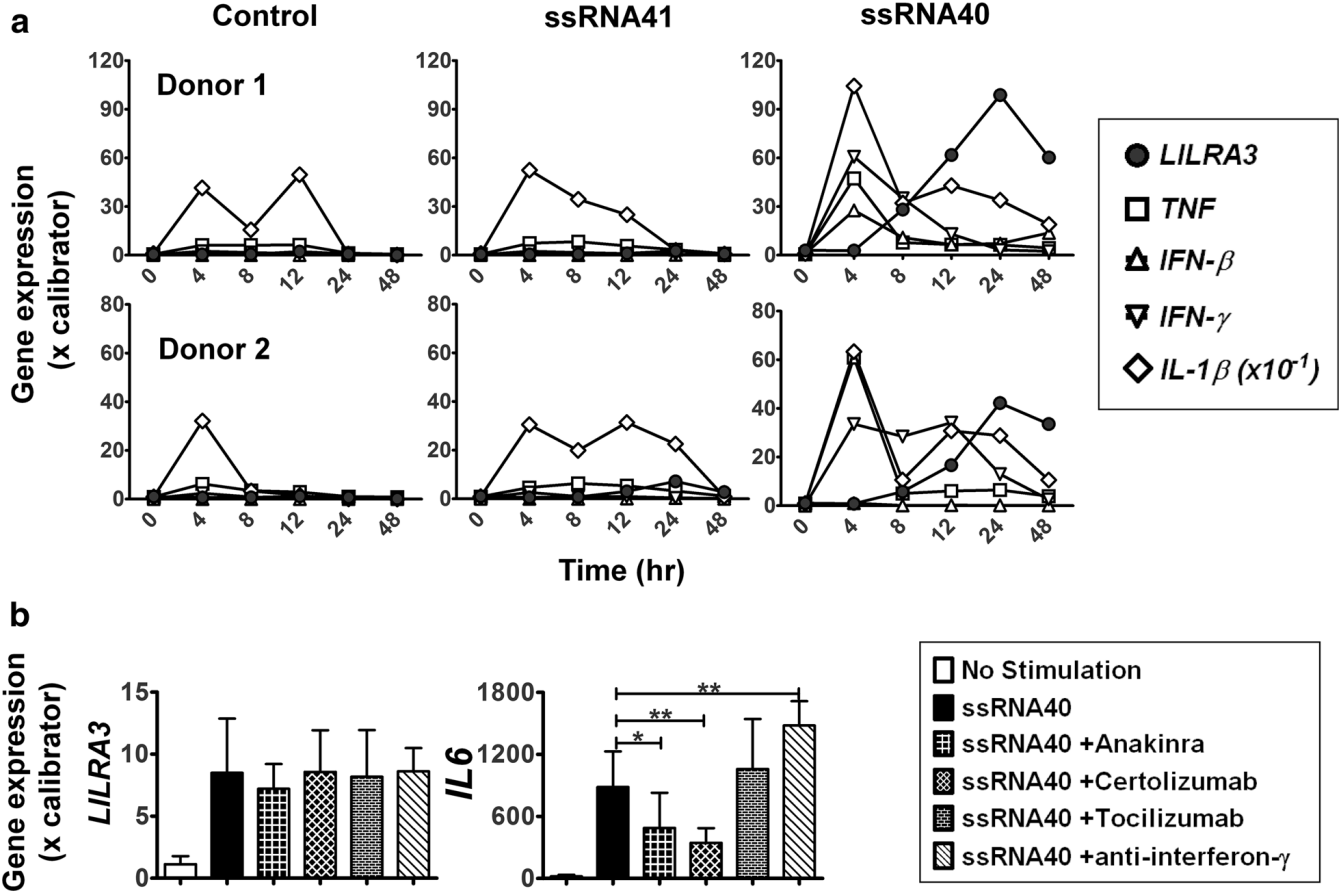
Features of ssRNA40-induced *LILRA3* expression. **a** Kinetics of *LILRA3* expression was compared to other ssRNA40 inducible cytokines. PBMCs from two donors were stimulated with ssRNA40 or ssRNA41 and the RNA was harvested at various time points. qPCR for *LILRA3*, *TNF*, *IFNb*, *IFNg*, and *IL1b* expression, normalized to *RPLP0* and relatively quantified to calibrator cDNA (pooled cDNA from 10. **b** Effect of cytokine inhibition on *LILRA3* expression. PBMCs from two donors were stimulated overnight with ssRNA40 together with neutralizing agents anakinra, certolizumab, tocilizumab and IFN-γ neutralizing antibodies and analysed for *LILRA3* and *IL6* expression by qPCR. Repeated measures ANOVA was used to calculate difference of ssRNA40+ neutralization to ssRNA40 alone and results displayed as mean ± SD

In order to determine the time point at which *LILRA3* expression is the highest, we performed expression kinetics of *LILRA3* using ssRNA40 stimulation of PBMCs. We also looked at the expression of other cytokines major cytokines as a comparison. Whereas the expression of the other cytokines tested peaked early at 4 h after ssRNA40 stimulation, *LILRA3* expression only gradually increased to peak at 24 h, after which its expression declined (Fig. 2a). In order to test of other cytokines were responsible for the expression, we neutralized IL-1R, TNF, IL-6R and IFN-γ with their respective neutralization antibodies (Anakinra, Certolizumab, Tocilizumab and IFN-γ neutralization antibody) to see if they had any effect on *LILRA3* expression. No appreciable difference in ssRNA40-induced *LILRA3* expression was observed even though expression of *IL*-*6* was decreased in the presence of Anakinra and Certolizumab and increased in the presence of IFN-γ neutralization (Fig. 2b). In summary, LILRA3 expression by TLR-8 stimulation peaks at 24 h and is independent from the expression of other immune relevant cytokines.

### TLR8 regulation of *LILRA3* is abrogated in HIV infection

**Fig. 3:**
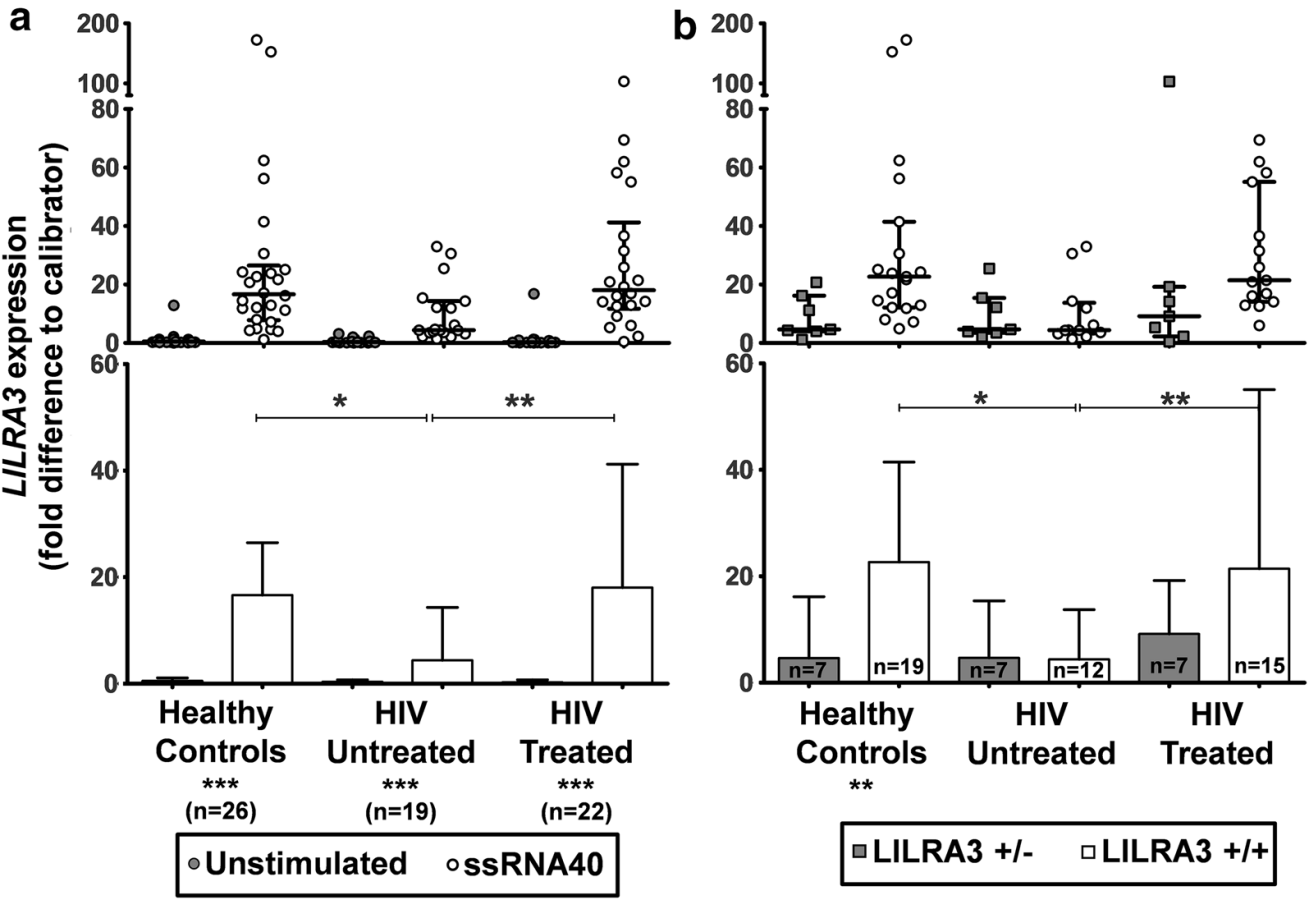
Analysis of ssRNA40-induced *LILRA3* expression in HIV. **a** Monocytes of healthy donors, HIV-untreated and HIV-treated patients were enriched using the Rosettesep system and stimulated overnight with ssRNA40 and analysed using qPCR for *LILRA3.* Wilcoxon matched-pairs signed rank test was used to compare intra-group unstimulated and ssRNA40-stimulated *LILRA3* expression. Kruskal–Wallis test was used to compare inter-group ssRNA40-stimulated *LILRA3* expression. Results displayed as median with interquartile range. **b** The values for the ssRNA40-induced *LILRA3* (without unstimulated controls) were segregated according to genotype (*LILRA3* ^+*/*+^ and *LILRA3* ^+*/*−^). Mann–Whitney test was used to compare intra-group *LILRA3* expression. Kruskal–Wallis test was used to compare inter-group *LILRA3* expression among *LILRA3* ^+*/*+^ donors. Results displayed as median with interquartile range (*p < 0.05; **p < 0.01; ***p < 0.001)

We performed real time PCR for *LILRA3* induction by 24-h incubation with ssRNA40 in genotyped HIV untreated patients, treated patients and healthy controls. Only samples of donors with at least one copy of the *LILRA3* gene were analysed. Since monocytes are the major producers of LILRA3 and the percentages of monocytes could vary widely between persons, we performed the stimulation on isolated monocytes so that the results would not influenced by monocytic counts. Intra-group Mann–Whitney test between unstimulated control and ssRNA40 stimulation revealed that all three groups had a significant upregulation of *LILRA3* expression upon ssRNA40 stimulation (Fig. 3a). However, Kruskal–Wallis with Dunn’s multiple comparison test between the stimulated samples revealed that *LILRA3* expression in untreated HIV patients was significantly reduced compared to healthy controls, which is partially restored in treated patients (Fig. 3a). Interestingly, segregation of the patient and control groups into *LILRA3*^+*/*+^ and *LILRA3*^+*/*−^ revealed higher *LILRA3* expression among the *LILRA3*^+*/*+^ in healthy controls and HIV treated patients, although it was only significant among the healthy controls (Fig. 3b). Even among the *LILRA3*^+/+^ samples, the HIV-untreated group had significantly lower *LILRA3* expression than the healthy group and HIV-treated group.

### HIV infection selectively abrogates ssRNA40, but not LPS, activation of *LILRA3*

**Fig. 4:**
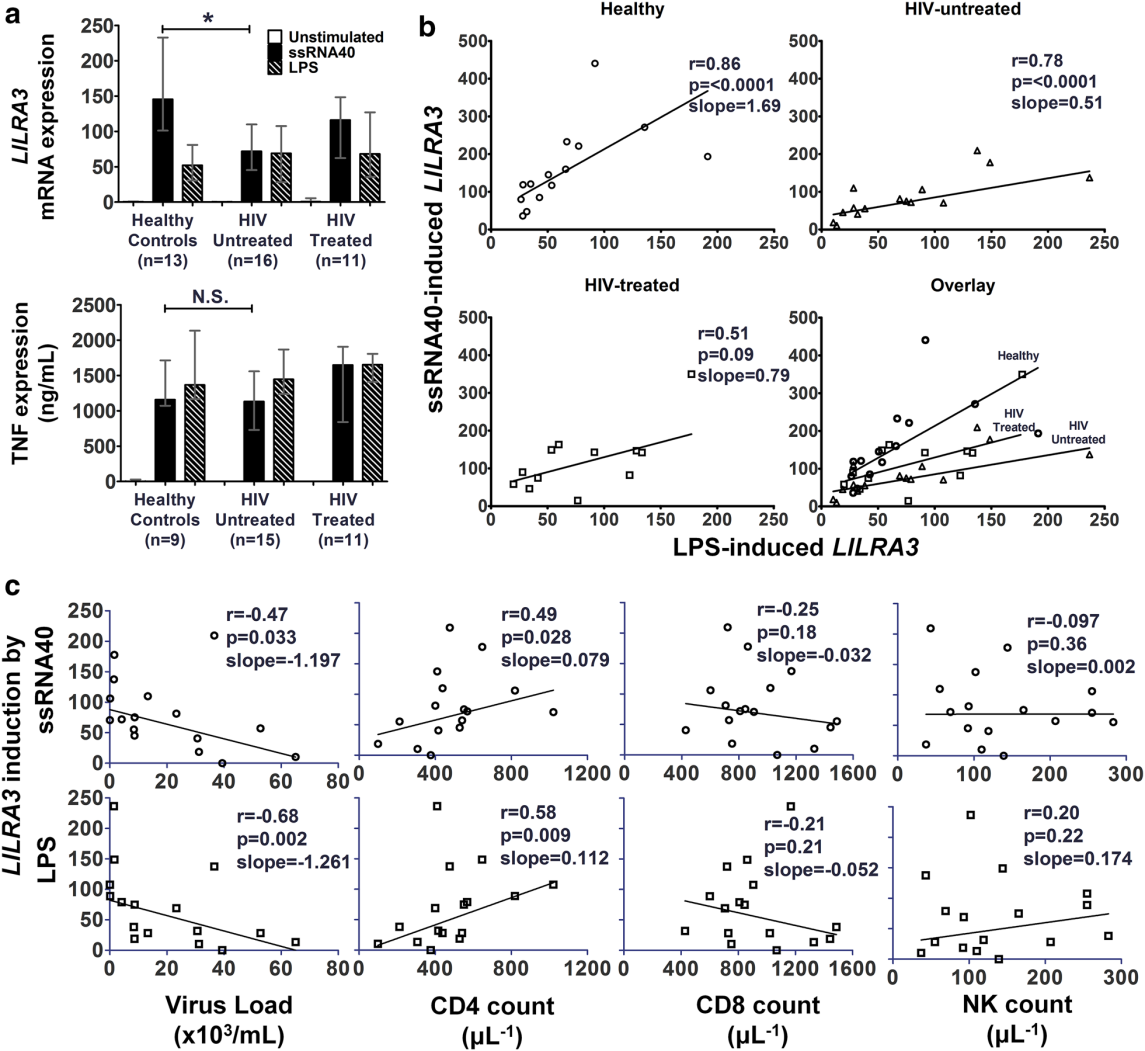
Relationship between ssRNA40 and LPS-induced *LILRA3*. **a** Monocytes of healthy donors, HIV-untreated and HIV-treated patients were enriched using the Rosettesep system and stimulated overnight with ssRNA40 and LPS. *LILRA3* expression was analyzed using qPCR and TNF expression was analyzed using ELISA. Kruskal–Wallis test was used to compare inter-group ssRNA40-stimulated *LILRA3* and TNF expression. *Bar graph* depicts median with interquartile range. **b** Correlation analysis of ssRNA40 versus LPS induced *LILRA3* expression. Two-tailed spearman analysis was used to calculate correlation. Non-linear regression straight line robust fit was used to calculate the trend *lines*. **c** Relationship between ssRNA40/LPS induced *LILRA3* expression and disease parameters in HIV-untreated patients. One-tailed Spearman analysis was used to calculate correlation between *LILRA3* expression and virus loads, CD4 and CD8 T-cell and NK-cell counts in HIV-untreated patients. Non-linear regression straight line robust fit was used to calculate the trend *lines*

As LPS seems to also induce *LILRA3* (Fig. 1a), albeit more weakly, we compared if there were differences between the TLR8 and TLR4-mediated *LILRA3* production. We performed the same experiment as before, with a new stimulus LPS for each sample. The second cohort confirmed the trend seen in the first cohort, with HIV untreated patients expressing less ssRNA40-induced *LILRA3* compared to the healthy controls (Fig. 4a). However, after LPS stimulation, no significant difference was observed between healthy controls and HIV patients. Analysis of TNF expression via ELISA did not show a significant difference between healthy controls and HIV patients after both ssRNA40 and LPS stimulation (Fig. 4a). Correlation of ssRNA40 versus LPS induction of *LILRA3* expression gave striking differences. Among the healthy controls, there was a much higher expression of *LILRA3* induced by ssRNA40 in comparison to LPS, with a slope of 1.69 using linear regression (Fig. 4b). However, this trend was reversed in HIV-untreated patients, with *LILRA3* expression higher after LPS rather than ssRNA40 stimulation, with a much more gradual slope of 0.51. Treated patients gave an intermediate trend, with a slope of 0.79. Correlation analysis showed a statistically significant relationship between ssRNA40 and LPS induced *LILRA3* expression for healthy donors and HIV-untreated donors.

### Both ssRNA40 and LPS-induced *LILRA3* expression correlates with viral load and CD4 counts

Since we believe that LILRA3 can be helpful in a virus infection due to its ability to induce proliferation in cytotoxic cell subsets [20], we hypothesize that expression of LILRA3 could indicate better disease status of the patients, i.e. lower viral load, higher CD4 and CD8 T-cell and NK-cell counts. We performed a one-tailed Spearman correlation analysis of both ssRNA40 and LPS-induced *LILRA3* expression with the abovementioned clinical parameters and observed that higher induction of *LILRA3* was significantly correlated with lower viral load of HIV-untreated patients. Virus loads were not analysed for treated patients because they were almost uniformly below 20 virus copies/mL. *LILRA3*-induction correlated positively with CD4 T-cell count in untreated patients (Fig. 4c), but not for treated patients (Additional file 2: Figure S2).

### Many immune activation genes are upregulated by LILRA3

**Fig. 5:**
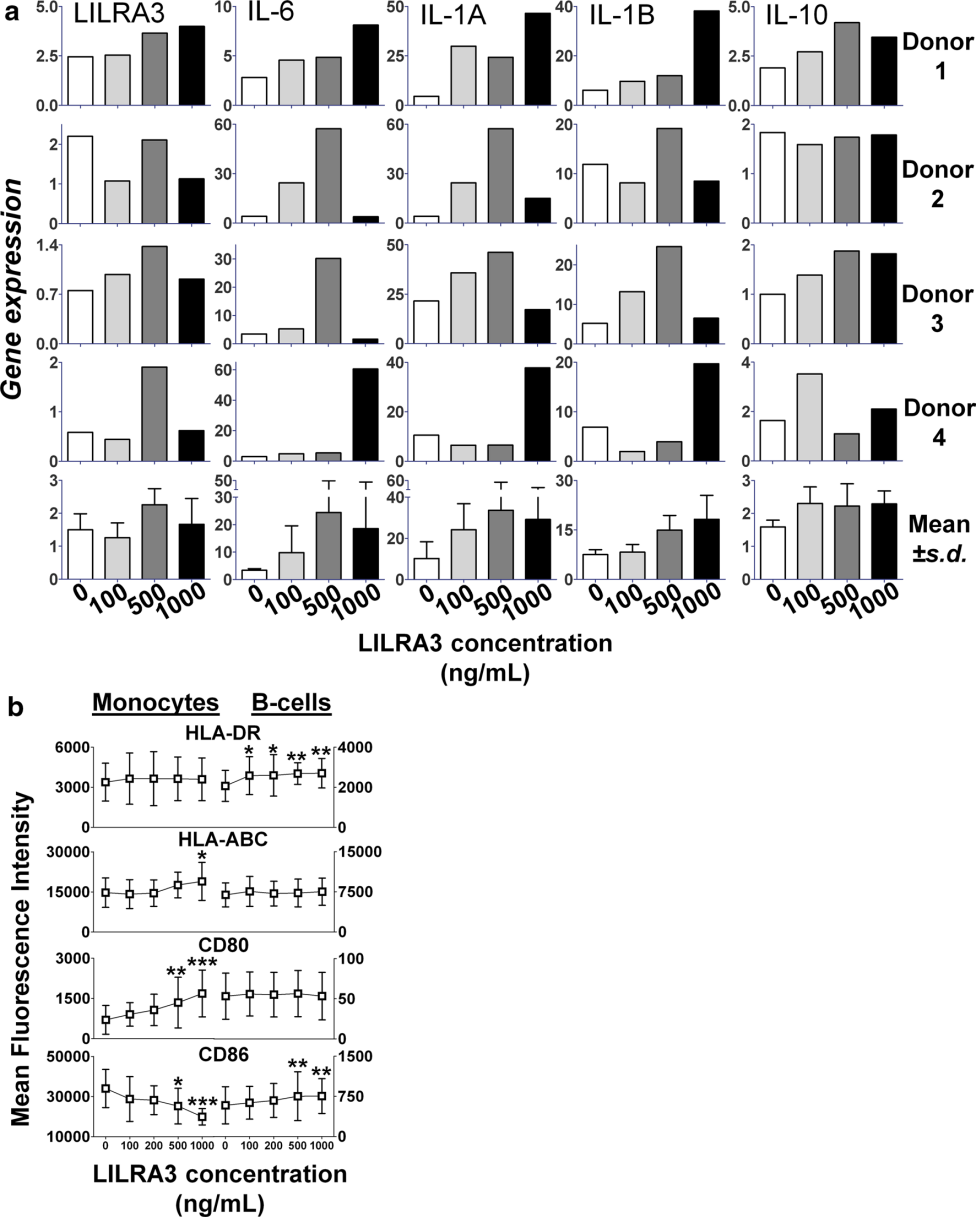
LILRA3 stimulation of cytokines and antigen presenting cells. **a** qPCR of LILRA3 induced gene expression of *LILRA3*, *IL*-*6*, *IL*-*1A*, *IL*-*1B* and *IL*-*10*. Due to the different optimal concentrations of LILRA3 for various donors, the gene expression of all four donors were shown, with the corresponding mean ± SD. **b** Effect of LILRA3 on the antigen presentation mechanism of monocytes and B-cells. PBMCs were stimulated with varying concentrations of LILRA3 for 48 h and analysed by flow cytometry for expression of CD80, CD86, HLA-DR and HLA-ABC on monocytes (CD14^+^CD33^+^) and B-cells (CD3^+^CD19^+^). On monocytes, upregulation of HLA-ABC and CD80 was observed, whereas CD86 was downregulated. On B-cells, there was a slight but significant upregulation of HLA-DR and CD86. 1-way ANOVA with repeated measures Dunett post test was used to compare to 0 ng/mL LILRA3 control. Results expressed as mean ± SD from six donors. (*p < 0.05; **p < 0.01; ***p < 0.001)

To identify genes and pathways regulated by LILRA3, we analysed the gene expression signature induced by overnight incubation of PBMCs with recombinant LILRA3. Analysis of the microarray data revealed that among the top 50 genes upregulated by LILRA3, there appears to be a dominance of genes involved in immune activation, the foremost of which are the acute phase inflammatory cytokines IL-6 and IL-1α (Additional file 3: Figure S3A). The list of genes downregulated by LILRA3 however, paints a rather mixed picture, with a sprinkle of immunoregulatory genes like TREM2, CXCL11 and proteins involved in metabolic processes (e.g. CD36 and FABP4) or with less well known functions (e.g. PLBD1, LGALS2). Using gene set enrichment analysis (GSEA), we discovered a highly significant enrichment of several processes and pathways, among which chemotaxis, hematopoietic cell lineage pathways and genes involved in the pathogenesis of the autoimmune disorder rheumatoid arthritis (RA) had the highest number of annotated genes upregulated (Additional file 3: Figure S3B). Using realtime PCR, we confirmed the upregulation of *IL6*, *IL1A* and *IL1B* by LILRA3 (Fig. 5a). However, the consolidation of the results from the 4 different donors were confounded by the different optimum concentration of LILRA3 required for stimulation for different donors, with donors 1 and 4 needing at least 1000 ng/mL LILRA3, while donors 2 and 3 requiring 500 ng/mL of LILRA3, beyond which the stimulation is abrogated. Purification from a mock-transfected expression did not lead to an increase of IL6 production like the purified LILRA3 (Additional file 4: Figure S4). LILRA3 did not have much of an effect on *LILRA3* itself and on *IL10* (Fig. 5a).

### LILRA3 effects on the MHC and costimulatory molecules

Since LILRA3 binds to an undetermined ligand expressed on the surface of monocytes and B-lymphocytes [20, 27], we tested if there is a direct effect of LILRA3 binding in the regulation of the MHC and costimulatory molecules expression levels on these cells. On monocytes, incubation with LILRA3 selectively upregulated HLA class I and CD80 surface expression. Notably, CD86 was conversely downregulated, whereas there was no significant change in HLA class II expression (Fig. 5b). On B-cells, there was a selective upregulation of HLA class II and CD86. However, no significant difference in the expression levels of HLA class I and CD80 were detected.

## Discussion

TLRs and LILRs are two families of receptors which are widely and distinctly expressed on immune cells. Other members of the LILR family of receptors have been implicated in the control of TLR activity to several different bacterial infections (reviewed in [28]). The LILRs have also been shown play a role in viral infections like HIV [29–31]. We showed that ssRNA40, a TLR8-agonist, is the most prominent regulator of *LILRA3* expression among the TLRs, and that this was mainly mediated by the monocyte population. ssRNA40 is a uridine-rich single stranded RNA sequence derived from the U5 region of HIV-1. Although ssRNA40 is an activating ligand for TLR7 in mice, in humans it has been shown to stimulate TLR8 responses in TLR8, but not TLR7, transfected HEK 293 cells [32]. Therefore, in our system, the ability of TLR7 and TLR9 to stimulate *LILRA3* might be overlooked as the cell populations that express these receptors are underrepresented among the PBMCs. In human macrophages and dendritic cells, TLR3-ligand poly(I:C) has been shown to induce IFN-regulated genes, but not proinflammatory genes like TNF and IL6 [33], which might explain the missing IL6 response to poly(I:C) in our system.

Although *LILRA3* expression peaks after 24 h, neutralization of IL-1R, TNF, IL-6R and interferon-γ did not lead to an appreciable change in *LILRA3* (Fig. 2b), meaning that it is regulated independently of these cytokines. This could imply that *LILRA3* expression is regulated directly by TLR8 signalling pathways or by other indirect pathways/cytokines, for example type I interferons, which is as yet unknown. This should be of interest for further investigation.

In HIV-untreated patients, we observed a significant abrogation of the monocytic TLR8-induced *LILRA3* in comparison to the robust response of healthy controls, as well as a reconstitution of this response in treated patients (Fig. 3). It is crucial to note that in this study, we do not measure the constitutive *LILRA3* expression in HIV patients, but rather the strength of the *LILRA3* response to TLR4 and TLR8 stimuli. This is more reflective of the immune status of the patients, rather than the constitutive expression due to viral loads. Nevertheless, the strength of the *LILRA3* induction seems to be correlated with the viral load in untreated patients (Fig. 4c), suggesting a link between the strength of *LILRA3* induction and the controlling of the infection. This would be consistent with previous studies showing that the strength of the TLR3, -7 and -8 responses are more robust among individuals who remain seronegative even after repeated sexual exposure to HIV [34], and that a mutant TLR8 allele which is more “functional” than the wild-type allele confers protection against HIV disease progression [35]. Considering that conventional dendritic cells also express TLR8 and are demonstrated play a role in control of HIV [36–38], it would be interesting to analyse their LILRA3 production in future experiments.

In order to assess whether the abrogation of LILRA3 expression in HIV infection is TLR8-specific or a general dampening of the monocytic response, we looked at the LPS induction of *LILRA3* expression via TLR4 in a second cohort. ssRNA40-induced *LILRA3* correlated well with LPS-induced *LILRA3* expression across the three groups, suggesting that they act on similar signalling pathways to induce *LILRA3* (Fig. 5b). However, while TLR8 stimulation gave a more robust *LILRA3* response than TLR4 stimulation in healthy controls, this trend is paradoxically skewed towards LPS in HIV patients, who might actually benefit more from the TLR8-ssRNA response rather than the TLR4-LPS response. It is tempting initially to attribute this to immune dysregulation in chronically HIV-1 infected patients. However, the correlation between viral load and CD4 counts to *LILRA3* expression that was present under TLR8 stimulation was even more strongly correlated under TLR4 stimulation (Fig. 4c), suggesting that the strength of the LPS response is also a factor in containing HIV-1. It has been shown that sensitization of monocytes with HIV-1 or TLR8-ligands led to higher production of proinflammatory cytokines by subsequent stimulation by LPS [39]. One explanation could be that because the TLR8 response was exhausted in these chronically HIV-1 infected patients, another TLR(s) was used as a compensatory mechanism. When we analyzed TNF production after ssRNA40 stimulation, we did not observe any significant decrease in TNF expression in HIV untreated patients, suggesting other factors at play other than TLR-8 exhaustion.

At this stage, it is still unclear whether the robustness of the LILRA3 induction is a cause or an effect of improved viral control and we cannot rule out in our study that the stronger LILRA3 responses are due to a general healthier immune status of the patients with lower viral loads. However, in light of a recent study of the genetics of *LILRA3* deletion in HIV patients by Ahrenstorf et al. (in revision), HIV patients have higher percentages of *LILRA3*^−*/*−^ donors and there is a faster progression of the infection with decreasing gene dosage. Taken together, there is evidence to suggest that LILRA3 is helpful in immune control of HIV.

*LILRA3* expression level is confirmed to be dependent on its gene-dosage, with *LILRA3*^+/−^ donors expressing substantially less *LILRA3* than LILRA3^+/+^ donors (Fig. 4b). The untreated HIV patients had such depressed levels of *LILRA3* induction that both genotypes gave similar low levels of gene expression. This is in line with a previous study that revealed decreased LILRA3 expression in *LILRA3*^+/−^ rheumatoid arthritis patients [40]. However, it is unclear whether this is the result of haploinsufficiency or the deletion allele being dominant negative.

Previously, we have shown that LILRA3 can induce a host of proinflammatory cytokines. We also show now in this paper that LILRA3 can upregulate transcription of *IL*-*6, IL*-*1A* and *IL-1B*, but not *LILRA3* itself and *IL*-*10.* Previous studies have shown that IL6 have suppressive effects on TNF production in vitro and in vivo [41, 42]. As LILRA3 upregulates IL6, but not TNF, the IL6 expression might have had repressive effects on TNF production, which could explain why Lee et al. [27] saw a repression of LPS-induced TNF by LILRA3. Interestingly, two out of the four donors we stimulated with LILRA3 responded best at 1000 ng/mL, whereas the other 2 responded at 500 ng/mL, but not at all at 1000 ng/mL (Fig. 5a). This means that LILRA3 activation is under very tight control, with different donors having different activation thresholds. The abrogation of its stimulatory effects at too high concentrations is most likely a mechanism against overactivation.

LILRA3 can modulate the expression MHC and costimulatory molecules in monocytes and B-cells. Although monocytes are the major producers of LILRA3 (Fig. 1d), the as yet unknown receptor for LILRA3 was found on monocytes and it exerts its immune modulating properties via the monocytes [20, 27]. One possibility is that this is a mechanism for infected monocytes to alert nearby monocytes about the presence of virus particles in the vicinity.

Gene set enrichment analysis showed that LILRA3 regulates many genes involved in the pathology of RA (Additional file 3: Figure S3B). In the Han Chinese population, the genetic presence of LILRA3 is associated with increased risk of rheumatoid arthritis [40].

## Conclusion

Our data point towards a role of LILRA3 in virus immunity. ssRNA40 is the most prominent inducer of *LILRA3*, which is produced by monocytes. Although HIV-1 patients express significantly less *LILRA3* in response to ssRNA40, the LPS-induced LILRA3 remained unchanged. We also revealed that both TLR8 and TLR4 mediated *LILRA3* expression can be correlated with viral loads and CD4 T-cell counts in untreated patients and that LILRA3 can upregulate proinflammatory cytokines, as well as induce changes MHC and costimulatory molecule expression monocytes and B-cells. Based on our findings, LILRA3 could potentially be exploited in the treatment of viral diseases like HIV.

## Methods

### PBMC and monocyte isolation and culture

PBMCs were isolated using density gradient centrifugation by layering blood onto Biocoll (Millipore) and centrifugation at 1200×*g* for 20 min without brake. The resulting buffy layer was then washed twice with PBS before use in subsequent experiments. Monocyte enrichment was performed using the RosetteSep™ Human Monocyte Enrichment Cocktail (Stemcell Technologies). Positive enrichment of CD14^+^ monocytes was performed using CD14 Magnetic Particles (Becton–Dickinson). Cells were counted using trypan blue staining on a Neubauer hemocytometer and cultured at 37 °C with 5 % CO_2_, in RPMI 1640 supplemented with 100 U/mL penicillin, 100 μg/mL streptomycin, 2 mM l-glutamine (all Biochrom), and 10 % AB serum.

### RNA extraction and reverse transcription

Total RNA was extracted using Zymo Research Quick-RNA Microprep (Zymo Research), utilizing RNase-Free Dnase Set (Qiagen) for in-column genomic DNA removal. Reverse transcription was perform using High-Capacity cDNA Reverse Transcription Kit (Life Technologies).

### Quantitative real-time PCR

Expression levels of LILRA3, TNF, IL-1α, IL-1β, IFN-γ, IFN-β, IL-6 were normalized to the housekeeping gene 60S acidic ribosomal protein P0 (RPLP0) and relatively quantified using the 2^−ddCt^ method either to the unstimulated control or to calibrator cDNA. Primer sequences are listed in Table 1. Calibrator cDNA was obtained from PBMCs ficoll purified from pooled blood of 10 healthy donors. Real-time PCR was performed using the Maxima SYBR Green/ROX qPCR Master Mix (Thermo Scientific) on the ABI PRISM 7000 Sequence Detection System (Applied Biosystems). The thermal cycling conditions composed of an initial denaturation step of 95 °C for 10 min, followed by 40 cycles of denaturation (95 °C for 10 s), annealing and (60/57 °C for 20 s) and extension/detection (72 °C for 40 s).

**Table 1:** Real-time PCR primers

Gene	Sequence (5′–3′)		Annealing temp. (°C)	Length (bp)	Source
*RPLP0*	Fwd	TGCTTGATATCACAGAGGAAACTC	60/57	81	(1)
	Rev	CAATCTGCAGACAGACACTGG			
*LILRA3*	Fwd	AGGAGTGGGGACGTGACTT	60	202	(2)
	Rev	GGTCTGGCACGGATCTGTC			
*IFNB1*	Fwd	ATTCTGCATTACCTGAAGGCCA	60	141	(1)
	Rev	CCAGAGGCACAGGCTAGGA			
*IFNG*	Fwd	TCGTTTTGGGTTCTCTTGGCT	60	243	(2)
	Rev	TCTCCACACTCTTTTGGATGCTC			
*TNF*	Fwd	CTGGAAAGGACACCATGAGCA	60	237	(1)
	Rev	GGGCCAGAGGGCTGATTAGA			
*IL1A*	Fwd	AGAGGAAGAAATCATCAAGC	57	122	(3)
	Rev	TTATACTTTGATTGAGGGCG			
*IL1B*	Fwd	TCTTTGAAGCTGATGGCCCTAAA	60	188	(1)
	Rev	GAAGGTCTGTGGGCAGGGAA			
*IL6*	Fwd	GCAGAAAAAGGCAAAGAATC	60	178	(3)
	Rev	CTACATTTGCCGAAGAGC			
*LILRB1*	Fwd	TTCTATGACAGAGTCTCCCTCTCGGT	60	99	(4)
	Rev	AGTTTGCATCCATCCCTGTGACTG			
*LILRA1*	Fwd	ATCACAAAACAAGACTGCCTCACA	60	81	(1)
	Rev	AGGACCAAGCCAGCTATGCC			

(1) NCBI Primer Blast; (2) Primer Bank; (3) Sigma Aldrich KiqStart; (4) Primer Quest

### Samples

Permission for the study was obtained from the Institutional Review Board (IRB) of Hannover Medical School. Blood from HIV-1 chronically infected but untreated patients and HAART-treated patients with controlled viremia were obtained with written informed consent according to the Declaration of Helsinki from the HIV outpatient clinic of the Clinic for Immunology and Rheumatology, Hannover Medical School. Patient characteristics are listed under Table 2. Blood of healthy controls were obtained anonymously from blood donors at the Institute of Transfusion Medicine, Hannover Medical School as well as from volunteers from the laboratory.

**Table 2:** Patient characteristics

	Healthy controls	HIV untreated	HIV treated	p value
Cohort 1				
Number	26	19	22	
Mean age (SD)	43.50 (12.15)	42.53 (12.87)	48.86 (10.00)	>0.05
Male:female	21:5	13:6	19:3	>0.05
Cohort 2				
Number	13	16	11	
Mean age (SD)	43.00 (14.49)	47.50 (12.70)	50.64 (12.92)	>0.05
Male:female	10:3	10:6	9:2	>0.05

### TLR-agonist stimulation experiments

The Human TLR1-9 Agonist Kit (Invivogen) was used for the pilot study. 10^6^/mL PBMCs were stimulated for 24 h with 1 μg/mL Pam3CSK4 (P3C), 10^8^ cells/mL heat killed *Listeria monocytogenes* (HKLM), 1 μg/mL polyinosinic-polycytidylic acid (Poly I:C) in high molecular weight (HMW) and low molecular weight (LMW) forms, 100 ng/mL lipopolysaccharide (LPS), 100 ng/mL flagellin, 100 ng/mL synthetic diacylated lipoprotein FSL-1, 1 μg/mL iniquimod, 1 μg/mL ssRNA40/LyoVec and 5 μM CpG oligonucleotide ODN 2006. For the kinetics, PBMC were stimulated with ssRNA40/Lyovec and ssRNA41 and harvested for real-time PCR analysis at various time points up to 48 h. For the rest of the stimulation experiments, PBMCs or monocytes were stimulated overnight for 20–22 h with respective stimulants. In later samples, the supernatants from monocyte stimulation was analyzed for TNF production was analyzed using the TNF elisa kit (Biolegend). Anakinra (Sobi), Certolizumab (UCB) and Tocilizumab (Roche) were obtained as leftovers from clinical treatments and used to neutralize IL-1R, TNF and IL-6R respectively. Functional grade purified anti-human IFN gamma (eBioscience) was used to neutralize IFN-γ.

### Western blot to detect LILRA3 secretion upon ssRNA40 stimulation

Purified PBMCs from LILRA3 positive and deleted persons were cultured in Optimem +0.5 % AB-serum in the presence or absence of 5 μg/mL ssRNA40, using 3 × 10^6^ cells per well per 100 µL in a 96-well plate. After 48 h incubation, the supernatant was harvested, spun down at 13,000 rpm for 5 min in a microcentrifuge and run on a 4–12 % reducing SDS-PAGE gel (Life Technologies), and transferred onto a nitrocellulose membrane. LILRA3 antibody clone 2E9 (Abnova) was used for detection, using Peroxidase Conjugated Stabilized Goat Anti-Mouse IgG (H + L) (Pierce) as secondary antibody. After stripping the membrane with Restore Western Blot Stripping Buffer (Thermo Scientific), IL6 was detected using clone MQ2-13A5 (Biolegend) together with Peroxidase Conjugated Stabilized Goat Anti-Rat IgG (H + L) (Pierce). SuperSignal West Dura Chemiluminescent Substrate (Pierce) was used in conjunction with Versadoc MP4000 (Biorad) to visualize the bands.

### DNA extraction and *LILRA3* deletion genotyping

Genomic DNA was extracted from whole blood using Quick-gDNA MiniPrep kit (Zymo Research) according to manufacturer’s instructions. *LILRA3* deletion genotyping was done by PCR using reverse primer 5′-GACAGCAGATTCTAAAACAGTG-3′ for both genotypes; forward primers 5′-CCCCTGGAGCTCGTGG-3′ for the presence genotype and 5′-CATCTCGATCTGCCACTGACAC-3′ for the deletion genotype. PCR for both genotypes was performed in multiplex using Amplitaq Gold DNA Polymerase (Applied Biosystems) using the following cycling conditions on the Biometra T3 thermocycler: initial denaturation at 95 °C for 20 min, followed by 43 cycles of denaturation (95 °C for 10 s), annealing and elongation (62 °C for 2 min) and a final elongation step of 62 °C for 10 min. PCR products were analysed on a 1.5 % agarose ethidium bromide gel for the presence (1150 bp) and deletion (250 bp) genotypes.

### Generation of recombinant LILRA3

Mammalian LILRA3 was produced under a Lentivirus expression system. Briefly, *LILRA3* cDNA was amplified by PCR using Phusion High Fidelity DNA Polymerase (Thermo Scientific) from total cDNA of PBMC from a LILRA3^+/−^ donor using primers (Fwd: 5′-CGGACTAGTATGACCCCCATCCTCACG-3′; Rev: 5′- CGACTAGTTCA ATGGTGATGGTGATGGTG CTCACCAGCCTTGGAGTC) incorporating SpeI digestion site at both ends and His-tag sequence at the 3′ end. The amplified *LILRA3*-*His* cDNA was cloned into the XbaI site in the multiple cloning site of pRRL-CMV-MCS-IRES-GFP [51], and the sequence verified by sequencing (MWG Operon). The newly generated pRRL-CMV-LILRA3-His-IRES-GFP was used to transfect HEK-293T cells using the LENTI-Smart INT kit (Invivogen) according to manufacturer protocol. Virus particles produced were concentrated using PEG-it Virus Precipitation Solution (System Biosciences) and used to transduce fresh 293T cells to generate stable LILRA3-His expressing cell line. A stably transduced cell line was also produced using of pRRL-CMV-MCS-IRES-GFP for the mock purification control. Mammalian LILRA3-His or its equivalent mock purification was produced by culturing the transduced cells using CELLine AD 1000 bioreactor flasks (INTEGRA Biosciences) and purifying the recombinant protein using HisPur Cobalt Resin from supernatant adjusted to pH 8.2–8.5. Endotoxin level was tested using LAL Chromogenic Endotoxin Quantitation Kit (Pierce) and determined to be virtually endotoxin free at 5 × 10^−6^ EU/µg protein. LILRA3 purified from Baculovirus expression system was produced as described previously [20].

### LILRA3 stimulation experiments

For microarray analysis, 10^6^/mL PBMCs were cultured for 24 h in the absence or presence of 1 µg/mL baculovirus LILRA3 in a 25 cm^2^ suspension culture flask. The cells were pelleted and their total RNA isolated using the Qiagen RNeasy Minikit according to the manufacturer’s instructions. Microarray and data processing are detailed below and deposited in Gene Expression Omnibus (accession GSE61356). To confirm the microarray data, PBMCs were stimulated overnight with 100, 500 and 1000 ng/mL of mammalian LILRA3 and analyzed for *IL*-*6*, *IL*-*1A*, *IL*-*1B*, *IL*-*10* and *LILRA3* expression. For changes in B-cell and monocyte expression levels of MHC and costimulatory monecules, PBMCs incubated for 48 h with 0–1000 ng/mL baculovirus LILRA3 were stained with CD14-APC-CY7 (Biolegend), CD19-V450, CD3-V500, HLA-ABC-FITC or CD80-FITC, CD86-PE (all BD Bioscience) or HLA-DR-PE (Beckman Coulter), and analyzed via flow cytometry.

### Microarray experiment and data processing

Cy3-labeled cRNA was synthesized using the one color Quick Amp Labeling kit (Agilent Technologies) according to the manufacturer’s instructions. cRNA fragmentation and hybridization onto the Whole Human Genome Oligo Microarray V2 was carried out as recommended by the Agilent One-Color Microarray-Based Gene Expression Analysis Protocol V5.7. Data were analysed using the R software and several packages from BioConductor [43] and other sources. Preprocessing steps comprised background correction (“normexp”), quantile normalisation, probe summarisation, and log2 transformation. For the background correction, fitted intensities were calculated by the convolution of normal and exponential distributions to observed foreground and background intensities [44]. For a robust analysis, median values were calculated from replicate samples for each gene. Differentially expressed genes (DEG) were obtained by selecting genes with a minimal fold change of two and significant changes to untreated controls keeping false-discovery rate adjusted p values of 10 % according to the rank products method in order to apply robust statistics for small replicate sizes [45, 46]. In order to further functionally characterise the selected genes, GOstats for identifying gene ontology terms was applied [47]. A functional analysis of the selected gene list was done with SPIA, the signaling pathway impact analysis [48]. GSEA served for understanding the genome-wide expression of all genes [49] via cellHTS2 [50]. The data sets are available under GEO accession number GSE61356.

###  Statistical Analysis

All statistical analyses were performed using Graphpad Prism 5.

#### Abbreviation

LILRA3 leukocyte immunoglobulin like receptor A3

## Additional files

10.1186/s12977-016-0248-y Induction of *LILRB1* and *LILRA1* expression by a panel of TLR agonists.*LILRA3* expression was measured, using qPCR, as fold change to the unstimulated control from PBMCs stimulated for 24 hours.
10.1186/s12977-016-0248-y Correlation between *LILRA3* expression and CD4 and CD8 T-cell and NK-cell counts in HIV-treated patients. One-tailed Spearman analysis was used to calculate correlation. Virus loads were omitted because almost all treated patients had loads below detection limit.Spearman analysis was used to calculate correlation. Non-linear regression straight line robust fit was used to calculate the trend lines.
10.1186/s12977-016-0248-y Gene expression analysis of LILRA3 stimulated PBMCs. PBMCs from 5 healthy donors treated for 24 hours with or without 1 µg/mL LILRA3 was analysed for their gene expression using Whole Human Genome Oligo Microarray V2. (A) The top 50 up- and downregulated genes based on fold change in their expression levels between untreated controls and LILRA3 treatment. (B) and (C) GSEA map based on gene ontology for biological processes and KEGG pathway mapping respectively. Genes regulated by LILRA3 appears to be enriched for, among others, chemotaxis, rheumatoid arthritis and hematopoietic cell lineage pathways. Size of the nodes is representative of the number of annotated genes within the pathway/term that is regulated, whereas the connection thickness is representative of the number of overlapping genes between connected nodes.
10.1186/s12977-016-0248-y IL6 upregulation by purified mammalian his-tagged LILRA3, but not by a mock purification. PBMC stimulated for 24 hours by LILRA3 or an equivalent amount of a mock purification was analyzed for *IL6* production by qPCR.
