# Supplementary figures and images for: TLR8 regulation of LILRA3 in monocytes is abrogated in human immunodeficiency virus infection and correlates to CD4 counts and virus loads
Source: Retrovirology. 2016 Mar 12;13:15. doi: 10.1186/s12977-016-0248-y (PMC4788896; doi:10.1186/s12977-016-0248-y)

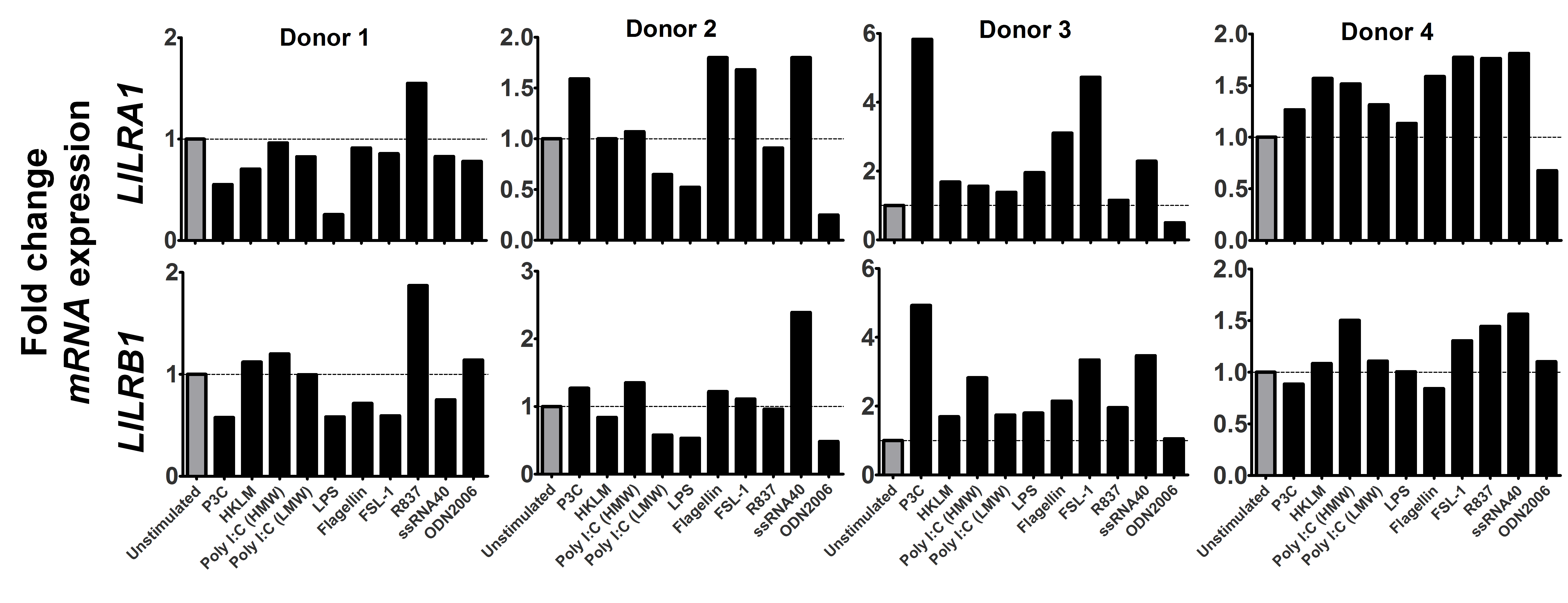

Supplement: Supplementary file 1 — 10.1186/s12977-016-0248-y Induction of LILRB1 and LILRA1 expression by a panel of TLR agonists.LILRA3 expression was measured, using qPCR, as fold change to the unstimulated control from PBMCs stimulated for 24 hours. [file 12977_2016_248_MOESM1_ESM.tif]

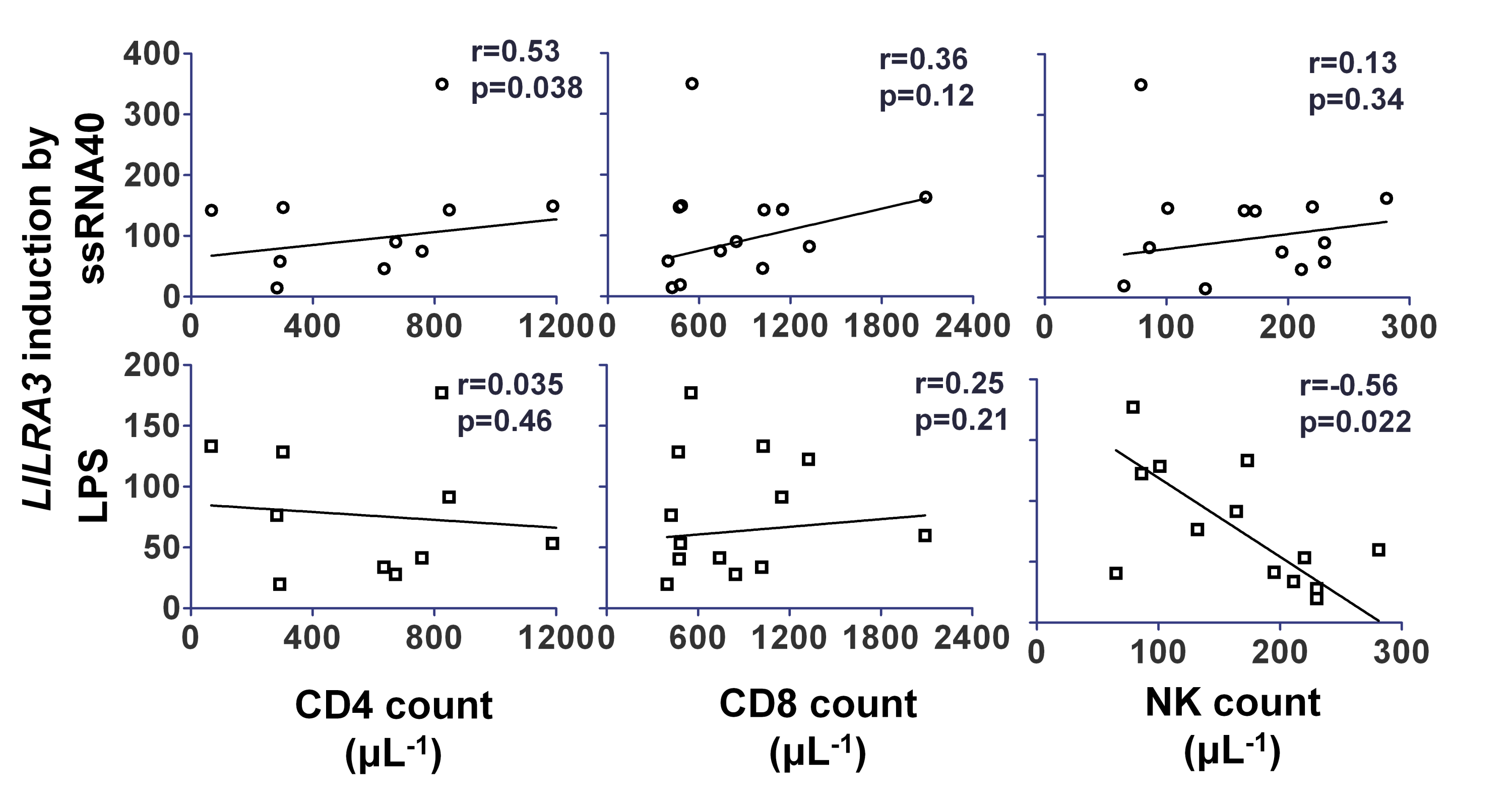

Supplement: Supplementary file 2 — 10.1186/s12977-016-0248-y Correlation between LILRA3 expression and CD4 and CD8 T-cell and NK-cell counts in HIV-treated patients. One-tailed Spearman analysis was used to calculate correlation. Virus loads were omitted because almost all treated patients had loads below detection limit.Spearman analysis was used to calculate correlation. Non-linear regression straight line robust fit was used to calculate the trend lines. [file 12977_2016_248_MOESM2_ESM.tif]

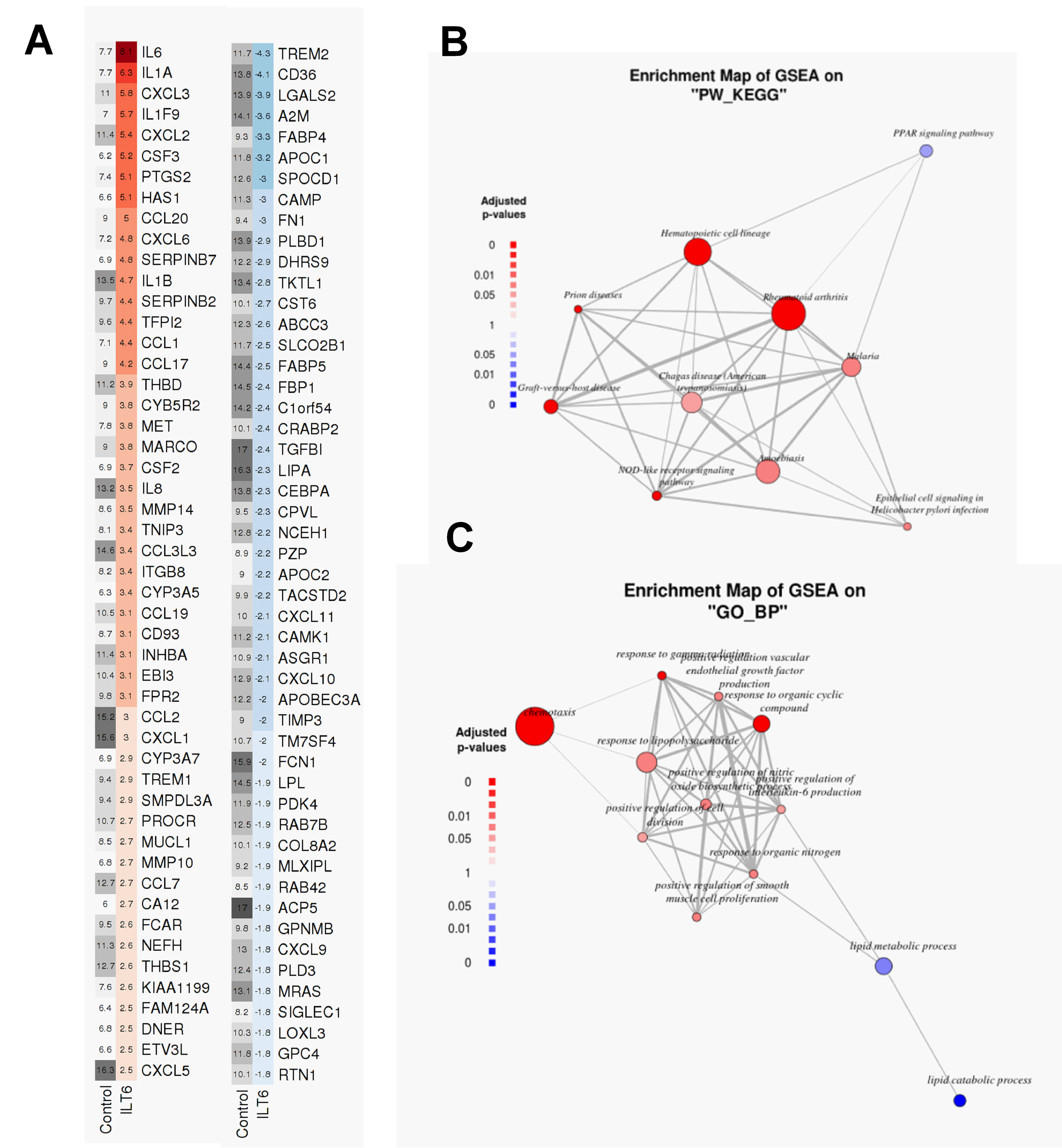

Supplement: Supplementary file 3 — 10.1186/s12977-016-0248-y Gene expression analysis of LILRA3 stimulated PBMCs. PBMCs from 5 healthy donors treated for 24 hours with or without 1 µg/mL LILRA3 was analysed for their gene expression using Whole Human Genome Oligo Microarray V2. (A) The top 50 up- and downregulated genes based on fold change in their expression levels between untreated controls and LILRA3 treatment. (B) and (C) GSEA map based on gene ontology for biological processes and KEGG pathway mapping respectively. Genes regulated by LILRA3 appears to be enriched for, among others, chemotaxis, rheumatoid arthritis and hematopoietic cell lineage pathways. Size of the nodes is representative of the number of annotated genes within the pathway/term that is regulated, whereas the connection thickness is representative of the number of overlapping genes between connected nodes. [file 12977_2016_248_MOESM3_ESM.tif]

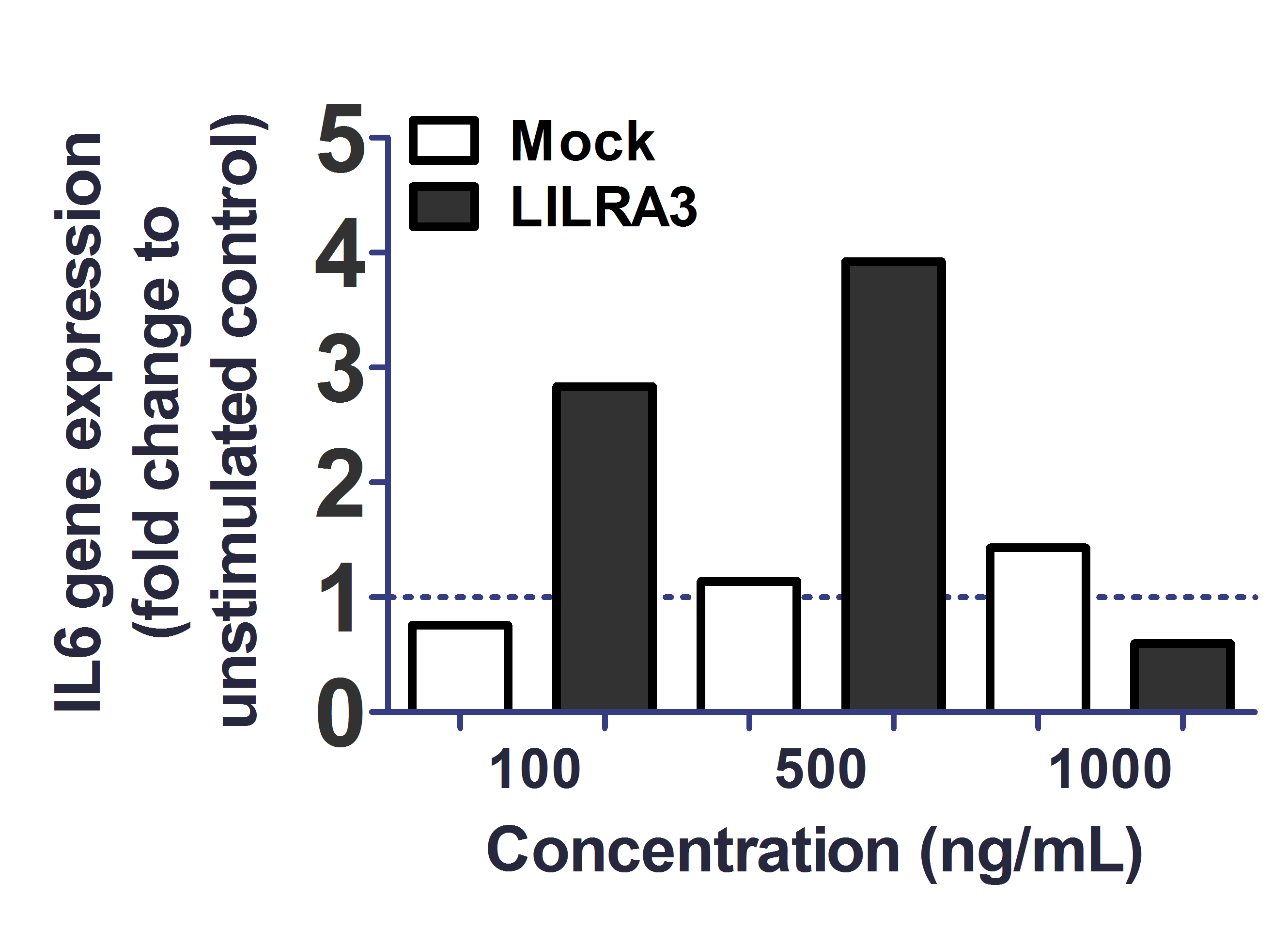

Supplement: Supplementary file 4 — 10.1186/s12977-016-0248-y IL6 upregulation by purified mammalian his-tagged LILRA3, but not by a mock purification. PBMC stimulated for 24 hours by LILRA3 or an equivalent amount of a mock purification was analyzed for IL6 production by qPCR. [file 12977_2016_248_MOESM4_ESM.tif]
